# An autonomous framework for interpretation of 3D objects geometric data using 2D images for application in additive manufacturing

**DOI:** 10.7717/peerj-cs.629

**Published:** 2021-08-10

**Authors:** Mohammad reza Rezaei, Mahmoud Houshmand, Omid Fatahi Valilai

**Affiliations:** 1 Department of Industrial Engineering, Sharif University of Technology, Tehran, Tehran, Iran; 2 Department of Mathematics & Logistics, Jacobs University Bremen, Bremen, Bremen, Germany

**Keywords:** Recurrent Neural Networks, Long-Short Term Memory cells, Convolutional Neural Networks, Feature recognition for additive manufacturing, Shape interpretation, Artificial intelligence in additive manufacturing, Additive manufacturing, Cloud based manufacturing

## Abstract

Additive manufacturing, artificial intelligence and cloud manufacturing are three pillars of the emerging digitized industrial revolution, considered in industry 4.0. The literature shows that in industry 4.0, intelligent cloud based additive manufacturing plays a crucial role. Considering this, few studies have accomplished an integration of the intelligent additive manufacturing and the service oriented manufacturing paradigms. This is due to the lack of prerequisite frameworks to enable this integration. These frameworks should create an autonomous platform for cloud based service composition for additive manufacturing based on customer demands. One of the most important requirements of customer processing in autonomous manufacturing platforms is the interpretation of the product shape; as a result, accurate and automated shape interpretation plays an important role in this integration. Unfortunately despite this fact, accurate shape interpretation has not been a subject of research studies in the additive manufacturing, except limited studies aiming machine level production process. This paper has proposed a framework to interpret shapes, or their informative two dimensional pictures, automatically by decomposing them into simpler shapes which can be categorized easily based on provided training data. To do this, two algorithms which apply a Recurrent Neural Network and a two dimensional Convolutional Neural Network as decomposition and recognition tools respectively are proposed. These two algorithms are integrated and case studies are designed to demonstrate the capabilities of the proposed platform. The results suggest that considering the complex objects which can be decomposed with planes perpendicular to one axis of Cartesian coordination system and parallel withother two, the decomposition algorithm can even give results using an informative 2D image of the object.

## Introduction

Today, the global market for products forces companies to compete for keeping their market position (Buckley, 2009). In this context, the ability to respond to the altering demands of the customers with customization in a relatively short time is a necessity (Gunasekaran et al., 2019). Consequently methods to create agility in companies are of much concern in this era and every progress which can improve the responsiveness of the companies facing high amount of altering demands and customizing the product features, is eagerly considered.

Beside the intense competition to attract customers, globalization has created a basis for better cooperation and collaboration between companies that can help them in their competition; the introduction of the internet of things (IoT) and cloud manufacturing, which has enabled companies to create pools of services and manufacturing resources to achieve cheaper manufacturing processes are two events that are going to reshape companies (Xu, 2012). On the other hand the large amount of data and complex decision making conditions, force companies to expand their reliance upon the artificial intelligence based tools (Kiron & Schrage, 2019; Davenport & Ronanki, 2018). Artificial intelligence provides companies with tools that ease the analysis of data and information in different phases of the production process and in some cases, it can replace the costly and error prone human agent. In fact, the rapid growth of knowledge in the field of cognitive science has improved the artificial intelligence tools to become more accurate mimicry of the human agent. As a result, they become more capable of replacing human agent in different domains. These paradigms shape the pillars of the new emerging industrial revolution, coined as industry 4.0 (Schwab, 2016; Vaidya, Ambad & Bhosle, 2018).

Another important progress in technology which is also at heart of the industry 4.0 is the additive manufacturing, or as known as its most popular technology, 3D printing (Schwab, 2016; Vaidya, Ambad & Bhosle, 2018). Additive manufacturing, as its old name “rapid prototyping” suggests, has been initially used as a technique for creating prototypes of products for further analysis during the product development process (Campbell, Bourell & Gibson, 2012). In fact, producing objects in a relatively short time and with high flexibility, was the first intention to use these methods, and after the introduction of less expensive methods, and increase in the demand side pressures on the companies, became their prominent advantage and justified their application (Mehrpouya et al., 2019; Thomas, 2013).

The combination of the power of cloud manufacturing and additive manufacturing methods can create a flexible, competitive and lower cost production ecosystem (Baumann & Roller, 2017; Hartman, 2020; Wang et al., 2019; Valilai & Houshmand, 2015). However, an important concern has to be addressed: although additive manufacturing methods have a great flexibility in producing objects without being restricted to geometric specifications, the selection of the proper additive manufacturing technology and proper additive manufacturing parameters are essential for the product pricing and decision making upon required techniques (Wang, Blache & Xu, 2017b). As a result it has to be decided autonomously that which product order should be produced by each of the available services in the cloud (Goodarzi et al., 2020). This is very essential when the additive manufacturing is considered to be accomplished by an XaaS approach in the cloud manufacturing demands (Valilai & Houshmand, 2015), where it has to be decided that whether each product be manufactured by each method and even the production parameters in each method have to be determined (Baumann & Roller, 2017).

The main objective of this paper is to propose an autonomous intelligent framework to interpret the geometry of the shape to create a basis for better integration of the design and manufacturing phases of the production process, reducing the constraints created on the design phase on behalf of manufacturing considerations (Wang et al., 2020; Wang, Blache & Xu, 2017a). In fact, the flexible structure proposed in this paper can create a basis for an autonomous recognition and expansion of the defined features based on the requirements of the system. In this framework, determining the best production technique to create the desired product can be accomplished autonomously by comparing the attributes of the features with capabilities of the available techniques.

An analysis of the studies about applications of machine learning in additive manufacturing shows that while there are studies which applied machine vision methods in solving issues of design and manufacturing processes in additive manufacturing (Wang et al., 2020; Scime & Beuth, 2018) and also there are studies which try to improve the integrity between these two processes by decomposing the part to smaller components (namely features) to make decisions upon them (Zhao & Guo, 2020; Ranjan, Samant & Anand, 2017), there is a lack of a flexible and autonomous structure, based on machine vision, to interpret the shape in terms of features for further use. In fact, studies like Zhao & Guo (2020) and Ranjan, Samant & Anand (2017) which try to interpret the geometry of the shape based on their features, omit the flexibility and learnability created by artificial intelligence based methods which makes the application of proposed methods problematic in applying them in cloud based platforms where nonprofessional customers want their products to be produced using simple 3D designs or even 2D pictures. On the other hand, studies like Wang et al. (2020) and Scime & Beuth (2018), which try to apply artificial intelligence based methods, overlook the need for autonomous intelligent methods in shape interpretation to make decisions upon production process, especially in cloud based platforms. The proposed framework in this paper is intended to be a basis to fill this gap.

The proposed framework is a multiplex of algorithms which decompose the shape into simpler parts and recognize, or categorize each part using two kind of neural networks separately. To illustrate the problem in better words the next section is dedicated to a review of some related previous works. In ‘Proposed framework’ the autonomous intelligent framework is explained in terms of its two main phases which are shape decomposition and recognition; then their connection is built up, such that they create one autonomous intelligent structure. Case examples shows the applicability of the framework applying it to two cases. In ‘Conclusion and Discussion’ the overall conclusion of the paper is explained and the future research potentials related to this paper are suggested.

## Literature Review

As stated earlier, as this paper aims to propose an autonomous framework for geometry interpretation, the dominant contexts of the Convolutional Neural Networks (CNN) and Recurrent Neural Networks (RNN) using Long-Short Term Memory cells (LSTM) are applied as mainstream artificial intelligence models. This work aims to use the capabilities of these models which can be found in literature studies like Ajit, Acharya & Samanta (2020) and Yu et al. (2019).

### Related previous works

An overall review of some related works in different approaches related to this study is presented in Table 1.

**Table 1:** Dominant research studies in the literature.

Research study	Research focus	Contribution
Biegelbauer, Vincze & Wohlkinger (2008)	Object recognition	A model for pose detection and object recognition based on the relative position of previously defined basic shapes
Wohlkinger et al. (2012)	Object recognition	A model to recognize 3D objects in CAD models based on 3D shape descriptors
Lake et al., (2017)	An overview of general approaches in artificial intelligence	An overview of the main trends in artificial intelligence methods and their shortcomings
Miyagi & Aono (2017)	Shape recognition	Applying a hybrid of CNN and LSTM to recognize 3D shapes represented as voxel grids.
Yin et al. (2020)	Shape recognition	Applying a modified version of CNN architecture for 3D shape recognition
Munguía, Ciurana & Riba (2009)	Build time estimation	A neural network for time estimation in additive manufacturing
Maidin, Campbell & Pei (2012)	Feature categorization	A design feature database to be used in product design in different disciplines
Conner et al. (2014)	Manufacturing method selection	A reference system for making decisions about the manufacturing method for a part considering customization, complexity and production volume
Moroni, Syam & Petrò (2014)	Production accuracy estimation	A simulation based model to estimate the accuracy of the manufactured part relative to the designed one
Piili et al. (2015)	Cost estimation of laser additive manufacturing	A model for the cost estimation of laser additive manufacturing of stainless steel
Mai et al. (2016)	Service composition in cloud manufacturing	A high level architecture for a cloud manufacturing system based on additive manufacturing for customized parts
Yao, Moon & Bi (2017)	Feature recommendation in design	A method providing feasible conceptual design solutions for inexperienced designers by recommending appropriate additive manufacturing design features
Shi et al. (2017)	Manufacturability analysis in a cloud based system	A three dimensional model to assess the manufacturability of a design in a cloud based additive manufacturing system
Wang, Blache & Xu (2017a), Wang, Blache & Xu (2017b)	Design for additive manufacturing in a cloud based system	Creation of a knowledge based framework to help users to apply design for additive manufacturing
Rudolph & Emmelmann (2017)	Automated order processing in cloud based additive manufacturing	Representing a cloud based platform to process customer orders automatically
Zohdi (2018)	Accurate tool path generation	A genetic algorithm based method for the tool path generation for a rapid free-form printing
Pham et al. (2018)	Shape categorization	A shape interpretation model to use CNN to categorize shapes due to their legitimecy of production
Zhao et al. (2018)	Nonplanar slicing and path generation	An algorithm to slice non planar surfaces based on flattening them into planar
Shi et al. (2018)	Feature based manufacturability analysis	A model for manufacturability analysis based on heat kernel signature
Chan, Lu & Wan (2018)	Cost estimation in cybermanufacturing	A framework for manufacturing cost estimation using LASSO regression
Zhao & Guo (2020)	Adaptive slicing based on type of features	An adaptive slicing method based on the type of the feature
Williams et al. (2019)	Quantitative manufacturing metrics estimation	A 3D Convolutional Neural Network to estimate manufacturing metrics from design
Simeone, Caggiano & Zeng (2020)	Service composition	A modular platform to improve the resource efficiency in a cloud of additive manufacturing services

More comprehensive reviews on the two main interests of this paper, including cloud based additive manufacturing systems and the application of artificial intelligence in additive manufacturing can also be found in Guo & Qiu (2018) and Goh, Sing & Yeong (2020) respectively.

### Gap analysis and research approach

Studies related to this paper are of the following categories:

• Studies with focus on shape, object or feature recognition or categorization. These studies are either of two groups:

– A group of these studies are trying to improve methods in artificial intelligence and machine vision in a general approach (Lake et al., 2017; Miyagi & Aono, 2017; Yu et al., 2019; Yin et al., 2020; Ajit, Acharya & Samanta, 2020). Such studies provide a good insight but their suggested methods should be modified to become applicable in an autonomous intelligent manufacturing framework. In fact some of their considerations, should be changed to be adjusted for applications in manufacturing.
– The other group of these studies include studies which are either adjusted for application in manufacturing or their view is inline with manufacturing concerns (Biegelbauer, Vincze & Wohlkinger, 2008; Wohlkinger et al., 2012; Maidin, Campbell & Pei, 2012; Yao, Moon & Bi, 2017; Wang, Blache & Xu, 2017a; Shi et al., 2018; Pham et al., 2018). The main problem with these studies is that they either omit artificial intelligence methods or apply methods which can not show a satisfying level of flexibility and learnability.

• Studies with focus on methods to estimate parameters of additive manufacturing or estimate production quality in additive manufacturing (Munguía, Ciurana & Riba, 2009; Moroni, Syam & Petrò, 2014; Piili et al., 2015; Shi et al., 2017; Zohdi, 2018; Zhao et al., 2018; Chan, Lu & Wang, 2018; Zhao & Guo, 2020; Williams et al., 2019). The viewpoint in these methods is restricted to calculate or improve special parameters or values, and the general interpretation of the geometry of the shape from manufacturing point of view is either omitted, which restricts the application domain of them and of their related works to special conditions and/or shop floor level decisions, or done using non-intelligent or partially intelligent methods. As a result, the latter group face the same issues as the second group of the first category.
• Studies with focus on the selection of manufacturing method or service composition in a cloud (Conner et al., 2014; Mai et al., 2016; Simeone, Caggiano & Zeng, 2020). These studies consider decisions in higher levels than the second category but they are either very general architectures of the cloud which omit some issues in the integration of the design and manufacturing or try to solve them using not or partially flexible solutions.

The studied literature in Table 1 shows that the most common application of artificial intelligence in the additive manufacturing deals with parameter fixation or output prediction, while geometrical interpretation as an initial step to connect customer view of demanded part with an automated production system has been neglected. In fact in some cases this issue has been overcame by explicitly defined shape models or other non flexible methods which can become confusing. The literature of design for additive manufacturing paradigm (Vaneker et al., 2020), emphasizes on some limitations on the design phase; these limitations create constraints on behalf of product customization and design by customers view (Wang, Blache & Xu, 2017a) while it is the duty of the production system to flexibly welcome the needs of the customers and to configure itself to meet them.

The main approach in this paper is to propose a framework to flexibly decompose an input shape, or even a 2D picture from the shape, taken from an informative viewpoint, into simpler shapes and to categorize them based on their similarity to previously categorized shapes. Although applying different neural networks is a common categorization method in different fields of study, including additive manufacturing, in most cases a neural network is responsible for the categorization from a scratch (Yin et al., 2020; Williams et al., 2019). The results of these models can get very accurate but as they are blind to the common, simpler building blocks of the shape, facing the complex shapes created by these building blocks usually gets problematic (Lake et al., 2017). The two fold framework proposed in this paper can overcome this problem by an initial decomposition before categorization or recognition.

This paper proposes a framework to interpret geometrical shapes (concerning additive manufacturing related ones) to analyze their production process automatically. As investigated in the literature, the automation of this step has not been of much concern. In fact this process is commonly accomplished by human agents and the human agent decides the production method or process; the dependence upon human agents makes the process time and money consuming and creates a discontinuity in the flow of data and information in an intelligent cloud based manufacturing ecosystem; but by using a flexible autonomous intelligent framework these costs can be reduced and a fully automated platform can be created.

Moreover, as the outputs of the proposed framework are general shape interpretations, they can be used as an input for parameter fixation models or intelligent systems to flexibly determine the best parameters to achieve better outputs; in doing so, this framework can ease the highly appealing shape categorization based quality improvement of the additive manufacturing parts. Finally, it should be said that the cognitive models such as one developed in Hummel & Biederman (1992) or gestalt perceptual apparatus (Wagemans, 2015) that are the main inspirations for the core idea of this paper, have to be of much greater considerations in engineering fields; because they create great insights into the human cognition, which can bring about many advantages, most importantly agility and flexibility.

## Proposed framework

As explained before, regarding the industry 4.0 revolution which demands the autonomous intelligence in production and collaboration in the manufacturing process to its full extent, having a framework that provides the system with the required flexibility and automation is a necessity. To create such a system, there has to be a flexible method to recognize the shape and to assign a production process based on this recognition. So one of the most important steps to have this system is to have an autonomous intelligent shape interpretation system.

**Figure 1:**
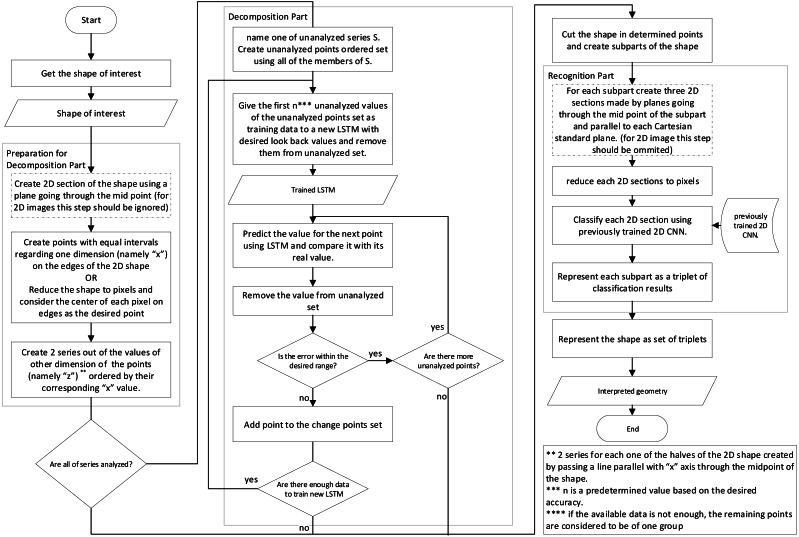
Flowchart showing the main structure of the proposed framework.

**Figure 2:**
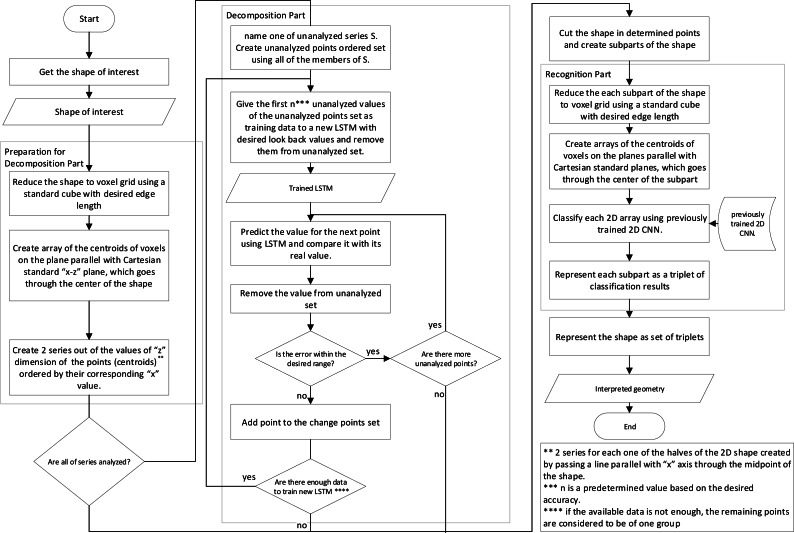
Flowchart showing the main structure of the proposed framework with another data preparation approach.

The interpretation of the shape is one of the most crucial steps in the production process, which takes part between conceptual design and manufacturing process; To create an autonomous intelligent shape interpretation structure, a two step framework is proposed that decomposes the shape into shapes which can be more easily recognized in the first step, and in the second step, each of the resulted shapes are recognized.The proposed framework uses simple information about the points, edges and plains of the object and as a result, can do analysis using the STL file format input or simple B-Rep model of shape or more complex CAD representation methods.  The main structure of the proposed framework has been depicted in Figs. 1 and 2. The two flowcharts show the same structure and their only difference is the data preparation phase for neural networks where two different approaches are proposed. The details of the framework, with respect to the second approach (Fig. 2) is discussed further in next parts of this section.

### Shape decomposition

The introduction of the design for manufacturing concept made an important contribution in the creation of the links between manufacturing processes and the design procedure (Wang, Blache & Xu, 2017a). This paradigm is advantageous in the cases where human agents play the key role, assuring that in the designed part the essential manufacturing factors has been taken into account; but considering the automatic manufacturing, specially from unstructured design data which is common in inverse engineering, it cannot be of any use. In fact there we need a method to interpret the shape, and there, the shape perception models of human cognition can be technically accurate source of inspiration.

The subject of the shape interpretation has been of interest before the introduction of the design for manufacturing concept and a great deal of literature in the process planning field is dedicated to the interpretation of the shape from a manufacturing point of view. In fact, there and in most other cases, the interpretation of the shape means the description of the shape in terms of a variety of some known shapes the attributes of which are known by the system and considering manufacturing point of view, can be produced by rather simple production processes (Ji & Marefat, 1997). In the manufacturing point of view, these are called geometrical features (Ji & Marefat, 1997). The basic idea of some of feature based shape interpretations can be found in perception models to describe human object recognition too. For example the constructive solid geometry (CSG) model (Ghali, 2008), is to some degree similar to the powerful perception model introduced by (Hummel & Biederman, 1992).

The classical feature based interpretation had some shortcomings which leaded to its abundance:

• Due to the classical artificial intelligent systems which were based on predetermined rules, there had to be rules to identify each feature, the creation of which was not an easy accomplishment.
• The complete definition of the features, required by those systems, made the recognition process restricted and the expansion of the system a difficult process.
• As they were completely based on classical manufacturing methods, their application in additive manufacturing methods become very limited.

**Figure 3:**
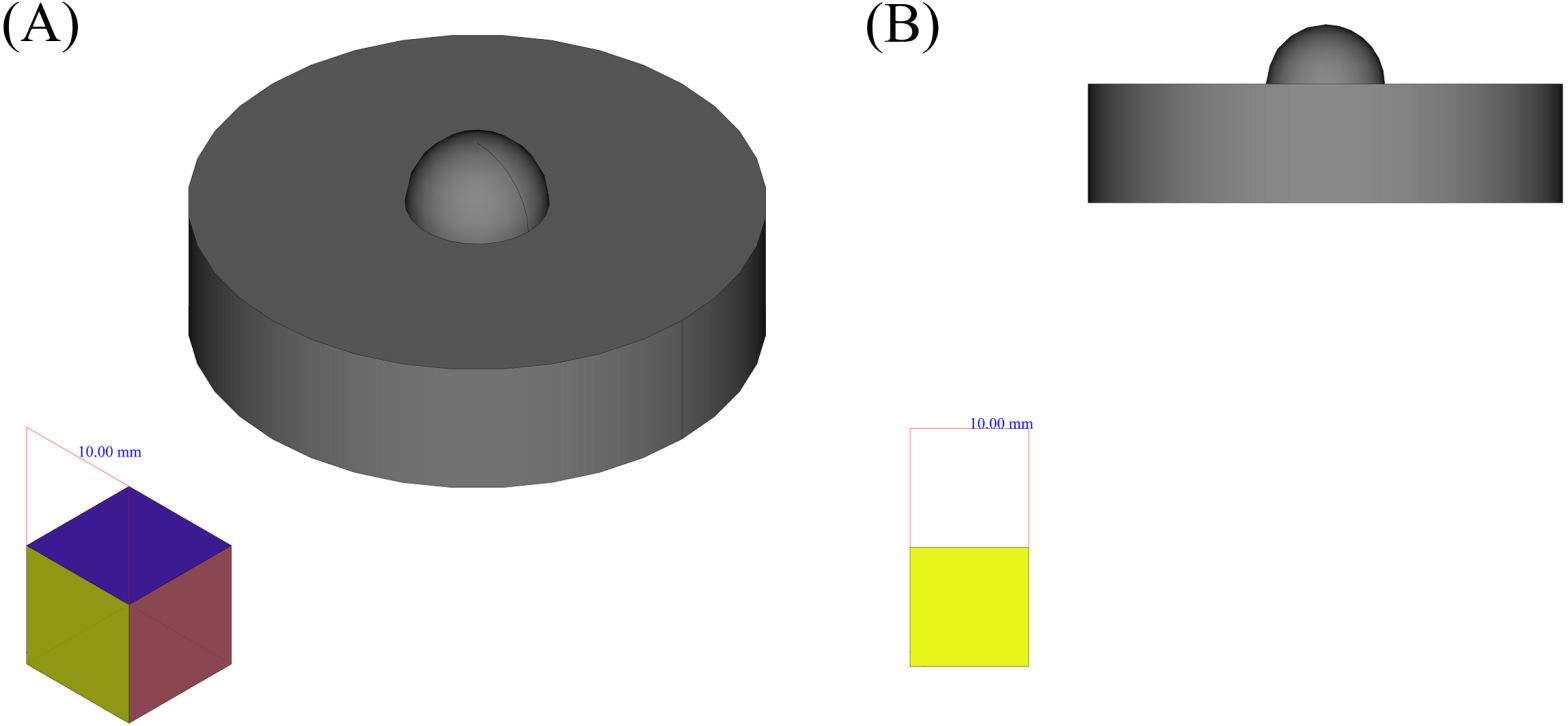
An example shape. (A) Isometric view. (B) Side view.

Although being problematic in some cases, geometrical features could be a good guide to interpret other shapes and to create knowledge, to analyze the complex shapes and produce them using known methods and considerations. On the other hand, facing the shape as a whole limits the power to use previous knowledge and in most cases, this knowledge would become vain. The method that can be used here, to keep the main concept of geometrical features, while making it more flexible and expandable is to decompose the shape into simpler shapes based on the conformity of the places of their outer edges points. As an example for this method, suppose the shape depicted in Fig. 3. It is obvious that the overall shape of the object is consisted of a cylinder and one half of a sphere. To decompose the shape, the only information used here is the placement of the vertices, faces and edges, *i.e.,* the information that is dealt with in ordinary 3D computer aided drawings, can be gathered through 3D scanning methods and is also available in STL file formats.

**Figure 4:**
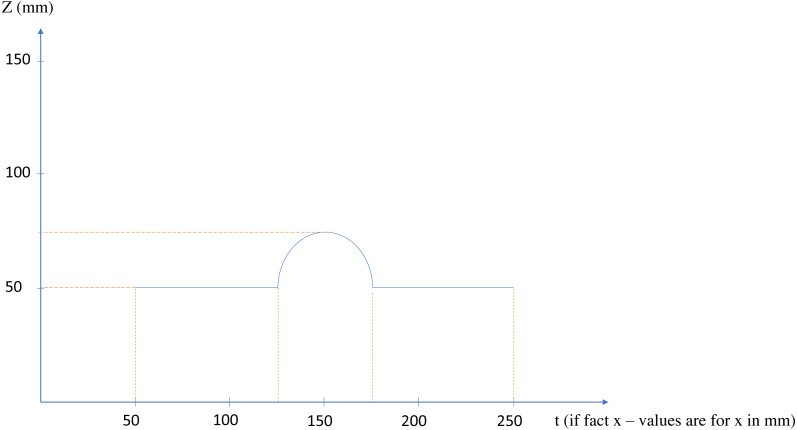
The *x* − *z* plane section of the shape of Fig. 3.

The main idea behind the method is to convert the type of one dimension from positional to temporal, and to consider corresponding position as a time series. For a better clarification, now suppose an imaginary scanner, scans the upper part of the shape, in the Cartesian *x* − *z* standard plane, and that plane passes through the center of the shape. If the scanner, scans one point per second and saves its relative position regarding the *z* axis, the placements of the scanned points will create a function of time as depicted in Fig. 4. Now, the critical points would be the points circled red, as they show the sudden changes in the behavior of the points or in better words, they show facing another connected shape; In these words, a simple shape, or geometrical feature in its new definition, would be a group of points, the behavior of which regarding a certain start point and direction can be determined by a pattern. This definition of the simple shape is in accordance with human perceptual apparatus, and to some degree in line with general gestalt rules (Wagemans, 2015), (especially continuity rule) and symmetry based definition of shape in Pizlo (2014).

In fact Fig. 4 is a 2D section of the overall lower face of the shape, on the Cartesian *x* − *z* plane (suppose that the midpoint of the shape is the reference point of the system), but considering the *x* axis as a time axis, provides us with a powerful, learner and flexible tool to recognize changes: RNN using Long-Short Term Memory cells (LSTM).

A simple change detection algorithm for changes in behavior using LSTM for a data set *DT* consisting of a series of data sorted according to time (or any other similar basis, *i.e.,* the position regarding one axis) is proposed in Algorithm 1.

 
Algorithm 1 LSTM based Change Detection Algorithm (namely ”LCDA”) 
 
  1:  Get as an input DT = {z1,z2,...,zn} which are the values of the series , the sudden changes 
     in which is of interest (or in other words values of the edge points on the z axis in the before 
     mentioned example). 
  2:  Get as an input DX = {x1,x2,...,xn} as the corresponding independent values for DT elements 
     for each of which, i.e. xi, the value of zi is of interest (or in other words corresponding values of 
     the edge points on the x axis in the before mentioned example, according to which the imaginary 
     point scanner of the example moves). 
  3:  Get as input parameters MTR as minimum size of data, W as the look-back or window pa- 
     rameter of LSTM and ME as minimum error coefficient. 
  4:  Get as input an LSTM architecture architecturelstm. 
  5:  Define set Changes = {}. 
  6:  Define variable BGN to represent index of the first training data for LSTM for each training 
     iteration. In better words, zBGN is the first data given to LSTM each time it is trained. Assign 
     the initial value as BGN = 1. 
  7:  Define variable TRSL to represent index of the last training data for LSTM for each training 
     iteration. In better words, zTRSL is the last data given to LSTM each time it is trained. Assign 
     the initial value as TRSL = MTR. 
  8:  Define variable ILD to represent placeholder for the data that needs to be checked. Assign the 
     initial value as ILD = TRSL. 
  9:  while |DT|− BGN > MTR and ILD < |DT| do: 
10:       Create training data set and name it TR such that TR = {zBGN,....,zBGN+TRSL} 
11:       Check whether the set Chx = {xi ∈ xBGN − 1,....,xBGN+TRSL such that: xi+1 ⁄= xi + 1} 
  is empty; if not add its members to Changes set. Set TRSL equal to the largest value in Chx 
    and go to step 17. 
12:       Create check data set and name it CD such that CD = {zILD−W−1,...,zILD−1}.  The 
     predicted value of zILD using LSTM in each iteration is of interest. 
13:       Create an LSTM and name it lstm with predefined architecture, architecturelstm and train 
     it using TR. 
14:       Use lstm to predict the values of TR.  Find the mean and standard deviation of absolute 
     value of difference between predicted and real values of TR. Name the results MER and SDTR 
    respectively. 
15:       Use lstm to predict the value zILD applying the CD as input. Name the result zpILD. 
16:       if |zpILD − zILD| > MER + (ME × SDTR) then 
17:            Update BGN such that BGN = TRSL. 
18:            Append ILD to the Changes set. 
19:            Set TRSL = TRSL + MTR. 
20:            Set ILD = TRSL. 
21:       else 
22:            Set TRSL = TRSL + 1. 
23:            Set ILD = TRSL. 
24:  Give the Changes set, which is the set of indexes showing the placement of the changes, as the 
     output. 
    

Considering the objects created from simple shapes put together in one direction (namely the direction of coordinate *z*) and can be separated using cutting planes perpendicular to axis *z* and parallel with *x* − *y* Cartesian standard plane, the proposed algorithm would be as described in Algorithm 2.

 
 
Algorithm 2 Simple LSTM based Decomposition Algorithm (namely ”SLDA”) 
 
  1:  Get as an input, the shape of interest and name it S. 
  2:  Get as an input parameter ml which determines the size of the largest edge of the shape to be 
     processed by the algorithm (which is determined relative to the computation power of the used 
     computer). 
  3:  Create a voxelgrid of S using an standard cube as unit (the edge length of which determines 
     the precision in contrasting different types of objects and bounded by the computing power 
     of the computer).  Name the resulted voxelgrid, V D which by definition would be:  V D = 
     [vd](cl × cl × cl) where vd would be 0 or 1 and cl is a value larger than or equal to the 
     mathematical ceiling of the ml value divided by the edge length of the unit cube (for complete 
     information on voxelization see Kaufman, Cohen & Yagel (1993)). 
  4:   Create matrix XZ = [vd(x,⌊cl∕2⌋,z)]. 
  5:  Define set Nx = {x′|∃z′ such that vx′z′ ⁄= 0}. Make Nx an ordered set by sorting it values. 
  6:  Find minimum and maximum values of z regarding each x in the indexes of nonzero elements 
     of XZ and create ordered sets minxz and maxxz out of these values sorted according to their 
     x values respectively. 
  7:  Create minxzi and maxxzi ordered sets by reversing the order in minxz and maxxz respec- 
     tively. 
  8:  Apply LCDA algorithm to minxz and minxzi separately; use Nx as the DX input for the 
     algorithm and append the resulted set of indexes together to create set Changes − 1 (change 
     the indexes in minxzi as to show the respective index of value in minxz).  Do the same for 
     maxxz and maxxzi and create the set Changes − 2. 
  9:  Set TChanges = Changes − 1 ∪ Changes − 2. 
10:  Give TChanges, which shows the placement of x dimensions of the changes from a simple shape 
     to another in the voxelgrid V D, as an output 
11:  Create Parts set from cutting the shape S using planes parallel with x − y plane which go 
     through points of S corresponding to detected changes in TChanges. 
    

**Figure 5:**
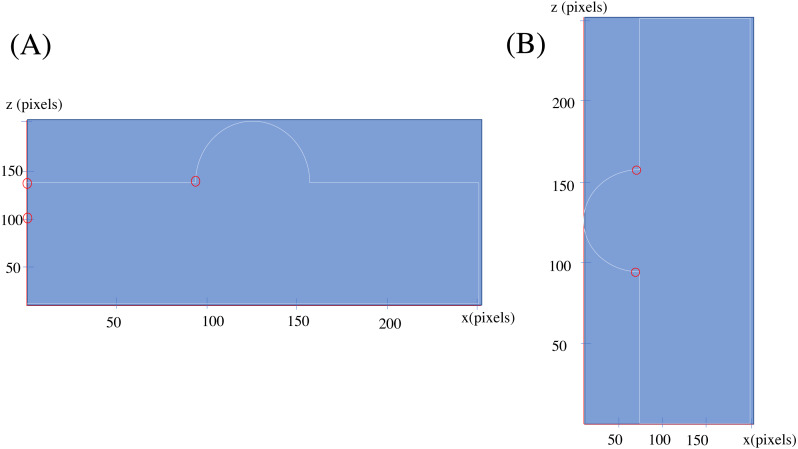
Changes found for shape in Fig. 3 in two orientation (A) Hemisphere component facing up. (B) Hemisphere component facing left.

By applying the SLDA algorithm into two rotations of the shape depicted in Fig. 3, and considering only Changes-1 set, the points circled red in Fig. 5 are resulted.

**Figure 6:**
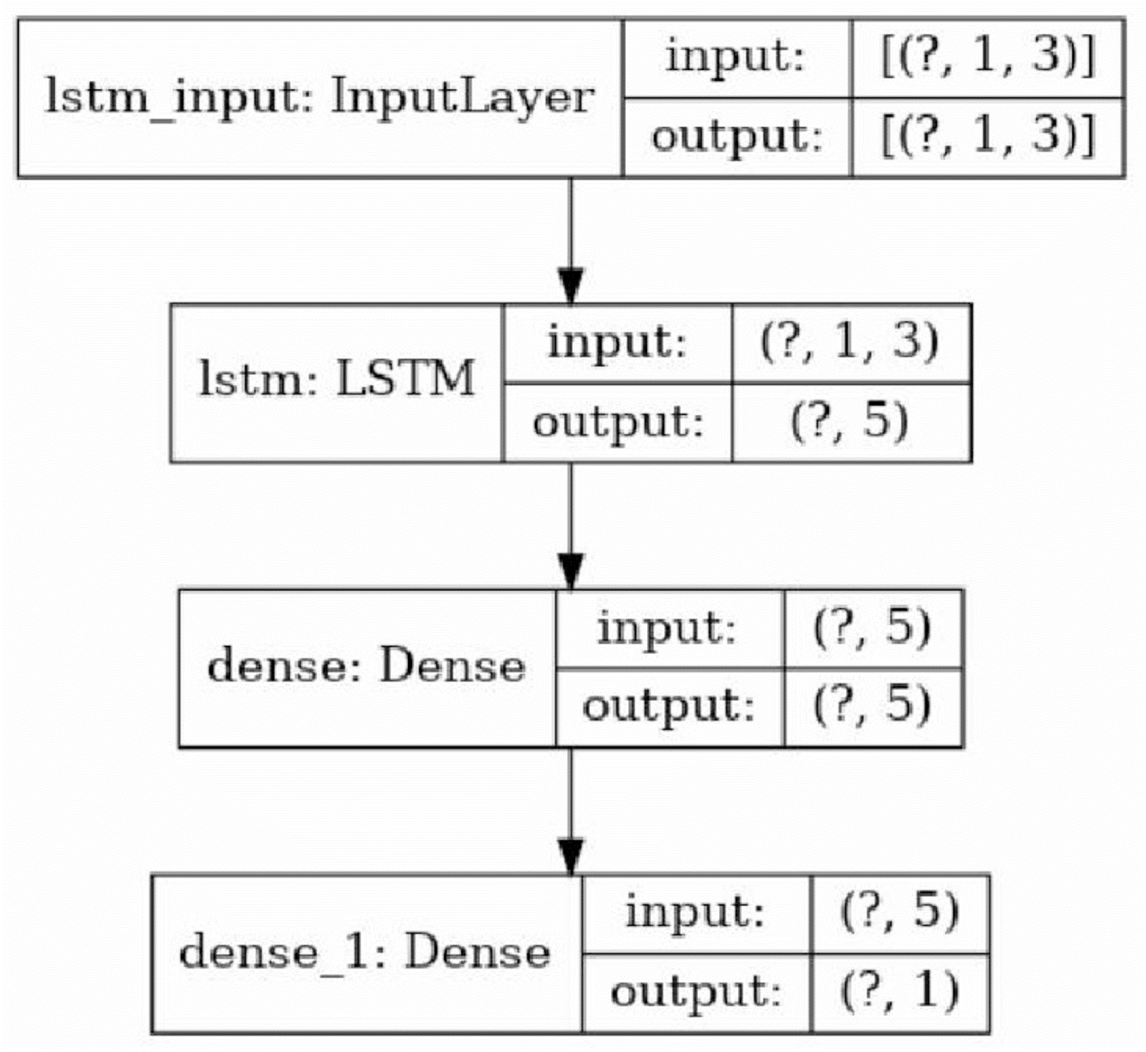
The architecture of the applied LSTM neural network. The question marks in the cells are due to the consideration of the overall data size as the first element of the array (the picture created using Keras^©^ library in python 3. 8.5^©^).

As is obvious, the resulted changes are not entirely similar: considering the two circled points in one row, as on the picture on the left side, shows an extra change. These errors are expected as the method is completely based on an statistical examination, checking the zero hypothesis of continuity of the previous pattern against the existence of a new one and as the neural networks have their own randomness in behavior, by fixing an architecture for our neural network and a coefficient of error in second step of LCDA algorithm (named *ME* there) an exchange of type 0 and type 1 errors in detection is decided. The results shown before are from assigning value 3 to *ME* and applying an LSTM neural network with an architecture shown in Fig. 6.

### Recognition algorithm

The decomposition process creates some simpler shapes to be recognized. The next step is to categorize these simpler shapes regarding their similarity with familiar cases. To do so, CNNs are the best candidate to use, as they can do the recognition process with a surprisingly high accuracy (Tong, 2018). Although there are 3D CNNs which could be used to carry out the recognition process in 3D shapes, the proposed framework uses a 2D CNN, taking sections passing through the center of the shape and parallel with each of the Cartesian coordinate system planes (*x* − *y*, *y* − *z*, *x* − *z*) as inputs. This method is applied because: 1Obviously i=1 and i = n are not of interest.
2This step is crucial to create functions out of points in one direction and an accurate definition of a series based on them.
3Although one check of changes can be enough, but as the changes may show off on the either side of the shape both *minxz* and *maxxz* are considered. Also as the change detection algorithm is prone to errors resulted from the batch of initial training data, it is suggested to perform algorithms on both orderings of the sets.
4In fact, two cutting planes going through the top and bottom of the corresponding voxels should be used and the shape between them should be omitted; this is due to to the use of voxelgrid.

• 3D CNNs are hard to train and are not as common as 2D CNNs, as a result finding good architectures for them is difficult.
• Creating 3D matrices as inputs for 3D CNNs is memory consuming
• In most cases, as the shapes usually have some kind of symmetry, these 2D sections are enough to interpret the shape
• If the input dimensions regarding three Cartesian coordinates have similar upper bound, and are interpreted by matrices of the same sizes, one 2D CNN would be enough.

**Figure 7:**
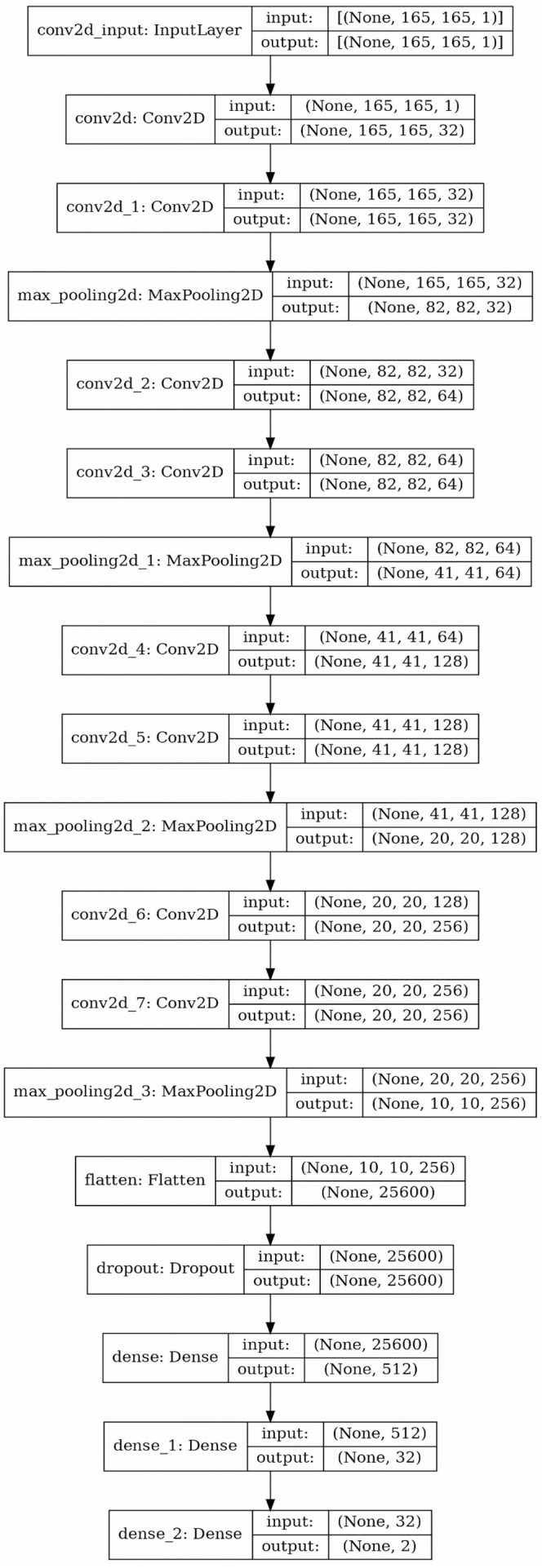
The structure of applied 2D CNN. As mentioned before the question marks in the cells are due to the consideration of the overall data size as the first element of the array (the picture created using Keras^©^ library in python 3.8.5^©^).

So a 2D CNN with an architecture depicted symbolically by Fig. 7 is trained and used for the recognition process. Using this neural network, the type of the object is determined by a triplet code, representing the type of each section.

### The main algorithm

Combining the mentioned steps, the overall framework would be Algorithm 3.


 
Algorithm 3 Main Algorithm 
 
  1:  Get as an input, the shape of interest and name it S. 
  2:  Create ordered sets Parts = {} and Types = {}. 
  3:  Get as an input parameter ml which determines the size of the largest edge of the shape to be 
     processed by the algorithm (which is determined relative to the computation power of the used 
     computer). 
  4:  Apply the SLDA algorithm and save the resulted shapes in the Parts set. 
  5:  for  each p in Parts do 
 6:       Rescale p such that its largest edge be of length less than or equal to ml . 
  7:       Rotate p such that its face with the largest area has a normal parallel to the axis z. 
  8:       Create a voxelgrid of p using an standard cube as unit (the edge length of which determines 
     the precision in contrasting different types of objects and bounded by the computing power 
     of the computer).  Name the resulted voxelgrid, V D which by definition would be:  V D = 
     [vd](cl × cl × cl) where vd would be 0 or 1 and cl is a value larger than or equal to the 
     mathematical ceiling of the m value divided by the edge length of the unit cube. 
  9:       Create  matrices  XY   =   [vd(x,y,⌊cl∕2⌋)]  ,   XZ   =   [vd(x,⌊cl∕2⌋,z)]  and  Y Z   = 
     [vd( ⌊cl∕2⌋,y,z)] where x,y,z ∈ [1,cl]. 
10:       Create an ordered triplet [XY,XZ,Y Z] using fore mentioned matrices. 
11:       Use  the  previously  trained  2D  CNN  to  determine  the  type  regarding  each  matrix  in 
     [XY,XZ,Y Z] and create [t(XY ),t(XZ),t(Y Z)] where t(a) is the type of a according to the 
     neural network. 
12:       Append the triplet [t(XY ),t(XZ),t(Y Z)] to the Types set. 
13:  Give Types set which shows the type of parts in terms of triplets, as output. 
    


This framework provides the compositionality and learnability at the same time; while the neural networks are used to find out the type of the building blocks of the shapes, which can be trained to recognize new basic shapes, the framework decomposes the main shape into simple shapes to be interpretable, without trying to recognize the type of the overall shape.

## Case examples

In this section to illustrate that how the proposed model works, it has been applied to two cases.

### General considerations

• In both cases CNN with architecture depicted in Fig. 7 has been applied. Activation functions in all layers except the last one were rectified linear unit and in the last layer it was set to Softmax. Optimization method was adaptive subgradient method (Duchi, Hazan & Singer, 2011).
• To prepare data to be used as an input for the CNN, the shape has been scaled, to make the length of the largest edge equal with 80 units. The cube with an edge length of 0.5 units is used to create voxelgrid of the shape. Then the shape has been put in a 185 × 185 × 185 array of voxels
• To train the CNN in both cases train data consisting of 4,000 randomly created circles or ellipses as curved or partially curved shapes and 4,000 randomly created rectangles, pentagons or hexagons as linear shapes has been used. The shapes were represented as 185 × 185 arrays. Uniformly distributed random integers have been generated and based on their value each shape for each group was created.
• For the first case, LSTM with architecture depicted Fig. 6 has been applied. Optimization method was RMSprop (Tieleman & Hinton, 2012). To initialize LSTM in each iteration, 40 points were used and the look-back value was set to 1.

### Case 1

**Figure 8:**
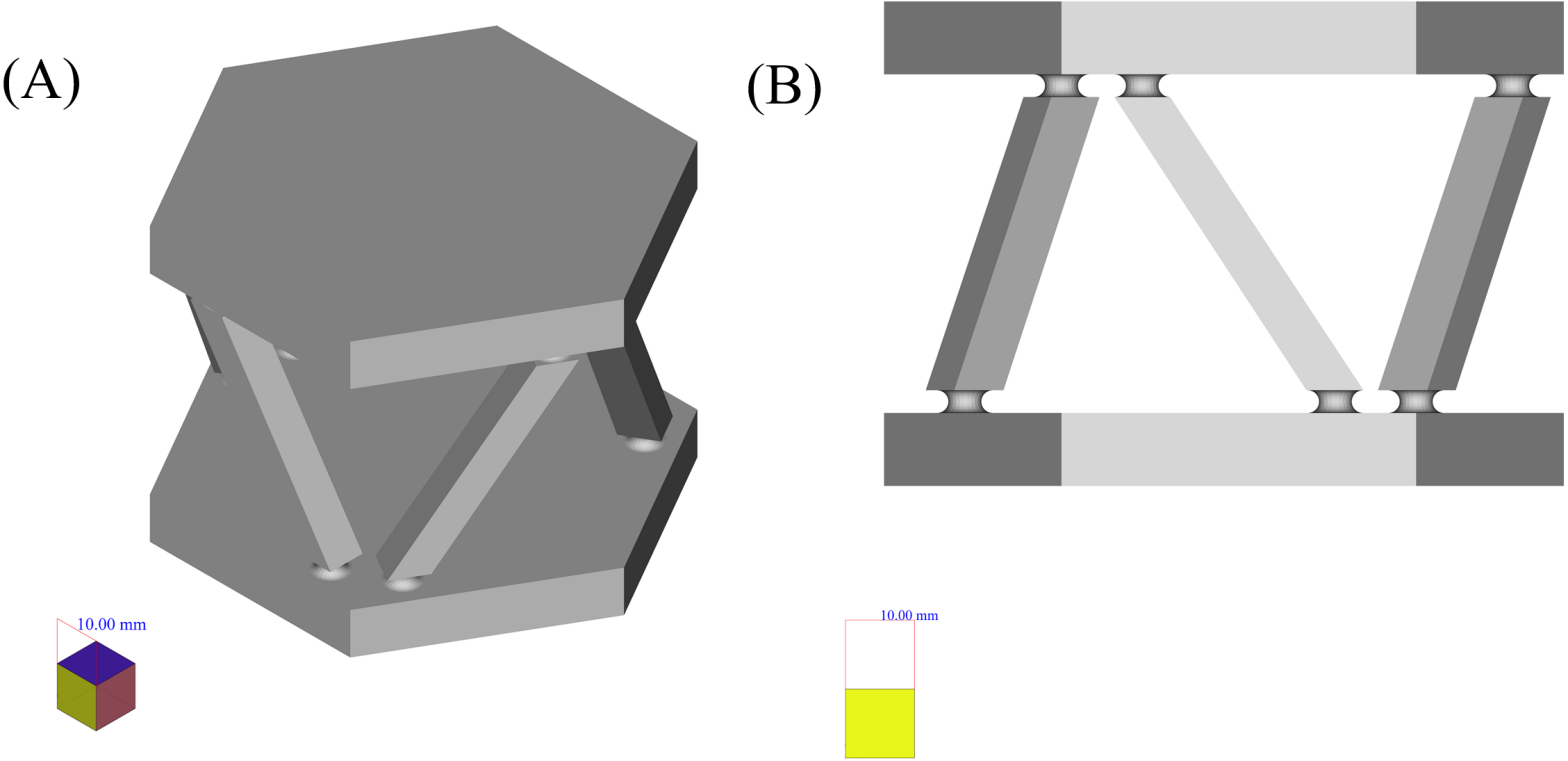
Six force sensor based on Stewart platform. (A) Isometric view. (B) Side view.

The first case, that has been chosen to be the input of the proposed framework, is the six force sensor based on Stewart platform depicted in Fig. 8. There were two reasons to choose this shape as a case:

• The shape is a symmetrical combination of different types of simpler shapes and is able to show the power of the framework in dealing with similar shapes.
• The shape is analyzed as a real case for the application of the manufacturing framework presented in Zhao & Guo (2020) where it has been decomposed using structured data available in high level CAD model and based on the curvature of its components, decision upon its manufacturing process has been made. Here it has been shown that how the shape analysis step can be done using less structured data and in a completely autonomous manner.

In the first step, to decompose the shape the framework can be applied in two ways:

1. Applying LCDA algorithm to the 2D mid section of the part as in SLDA algorithm. In this respect the SLDA is improved to act in a divide and conquer manner such that by detection of each change, a perpendicular slicing is done and the algorithm is applied to each of resulted parts.
2. Applying SLDA algorithm but instead of the mid section in step 4, the 2D image of the shape as a whole, on a plane, as depicted in the right hand side of Fig. 8 be used.

**Figure 9:**
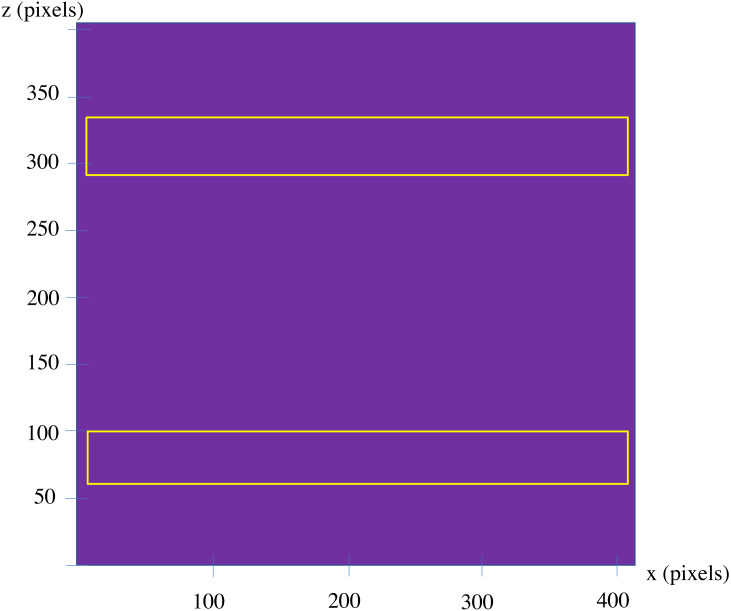
The first midsection of the part.

Here, both approaches are applied to the example, applying the first approach, at first. To apply the model, the shape has been scaled uniformly, to make the size of the largest edge of the shape, equal with 100 units. Then, the standard cube with the edge length of 0.25 units has been used to create the voxelgrid of the shape. By creating a midsection of the voxelized part as a whole, the 2D image depicted in Fig. 9 is resulted. Applying the LCDA, the step 11 of the algorithm, guarantees the separation of the two prismatic ends in a consequent manner.

**Figure 10:**
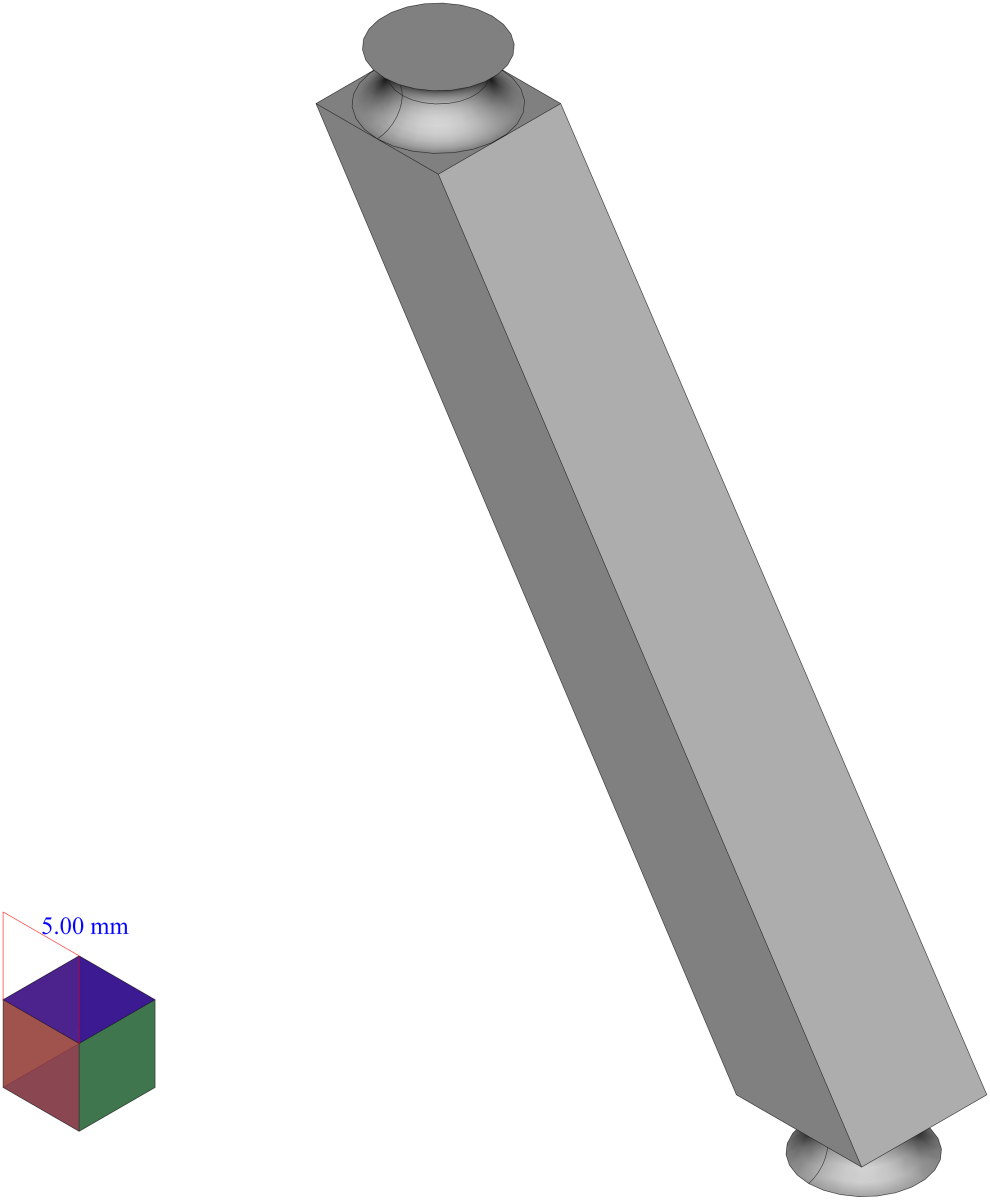
One of six remaining components.

As these two ends can not be decomposed further, six components with the shape depicted in Fig. 10 remain to be decomposed. Although the algorithm faces them separately (as it is blind to such similarities), only one of them is considered here (the others are faced in the same manner).

**Figure 11:**
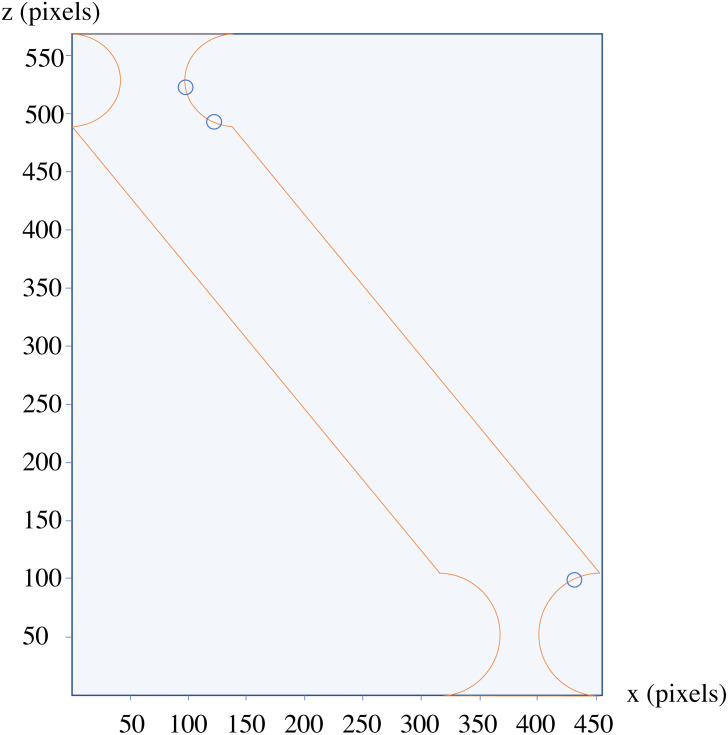
The results of the analysis of the component depicted in Fig. 10.

The analysis of the depicted components using only one side of the 2D section results in detected changes, which are circled blue in Fig. 11.

As mentioned before, to apply the second approach to analyze this example, the 2D image of the object as shown on the right hand side of the Fig. 8 can be used as an input to SLDA algorithm.

**Figure 12:**
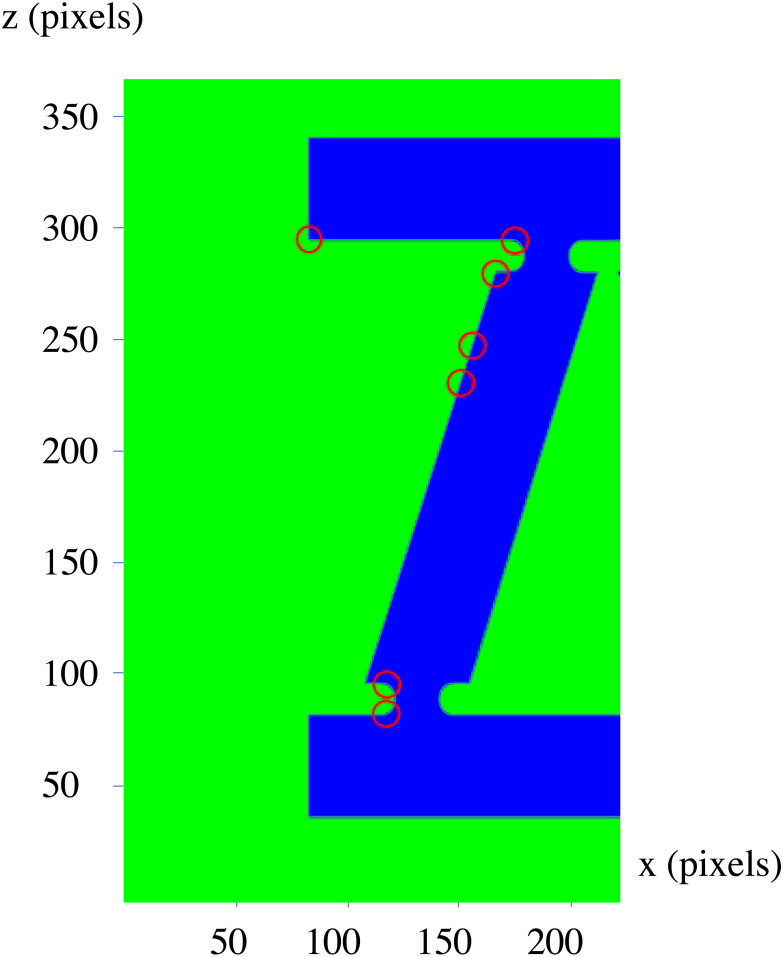
The results of the analysis of the 2D image of the shape.

Here a 2D picture of the shape with resolution of 3, 000 × 1, 500 pixels has been used. Applying the second approach, and only applying the algorithm for one side of the picture, the points which are circled red in the Fig. 12 are resulted.

In both approaches, more change points than the real ones are marked; as mentioned before, due to randomness in the behavior of neural networks, in these models a decision upon acceptance interval for a statistical examination should be done considering the errors type 0 and 1; as the larger errors in recognizing excessive points as change points, is much less important than error of not recognizing the real change points, the acceptance interval is configured for less error of not recognizing which results in excessive points, marked as change points. Another reason for the excessive change points is disruptions created by representation of the continuous lines by discrete pixels.

**Figure 13:**
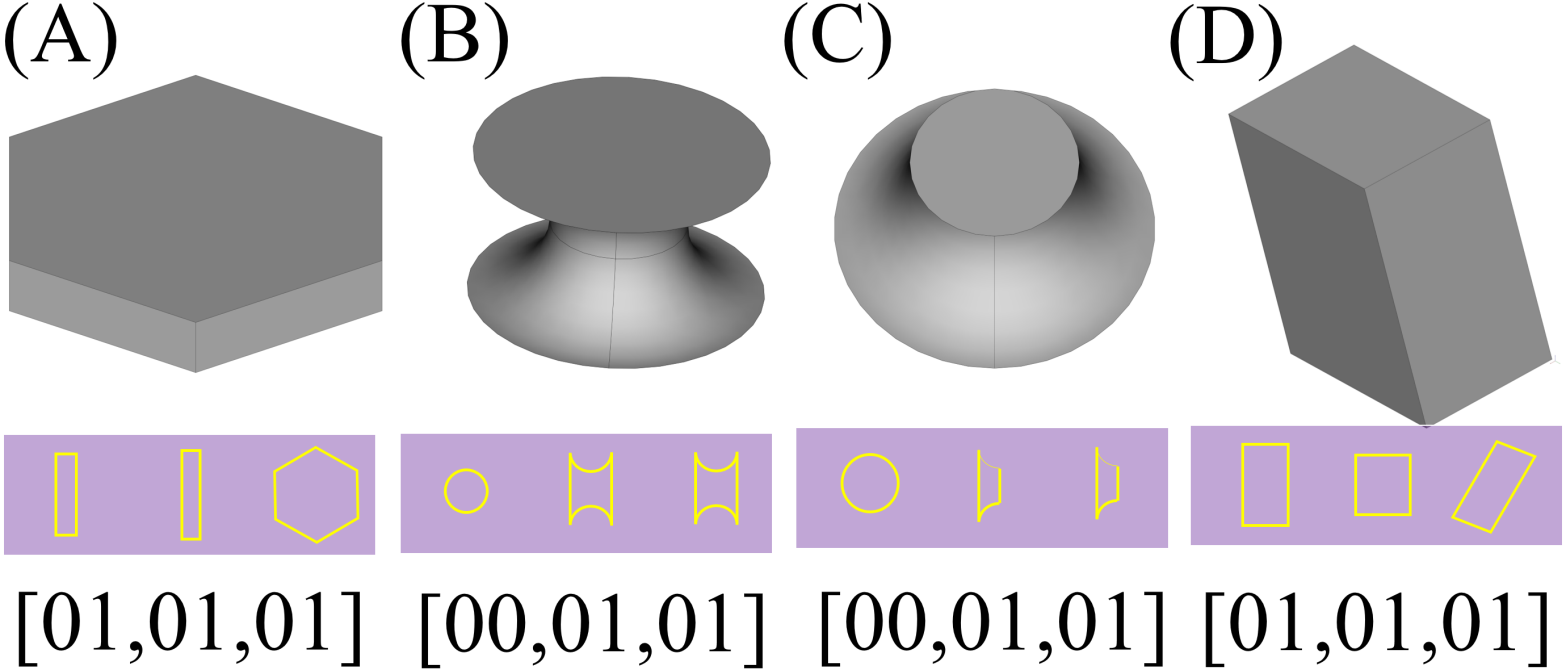
The 2D sections of the components. Pictures (A) to (D) depict one of each type of resulted components and their sections and the results of the recognition phase.

The next step would be the recognition of the shapes based on the presupposed standard. As mentioned before, the applied method is to create three 2D sections of the parts using planes parallel with each of the Cartesian standard planes which pass through the center of the shapes. In the first step, a preparation process as described in section ‘General considerations’, has to be conducted which results in matrices with standard sizes that can be used as an input to a 2D CNN with a fixed size of input layer. The resulted voxelgrid which can be interpreted as a 3D matrix can be sliced into three 2D matrices by fixing a number for each of its coordinate indexes; and as if these numbers be the middle of the maximum and minimum values of that index, the concerned 2D sections would be resulted. The resulted 2D sections for each shape is depicted underneath it in the Fig. 13 (The resulted matrices are depicted as 2D shapes in which the yellow pixels represent value 1 and other pixels represent value 0). Having the matrix representation for the three sections of concern, their type would be determined using a previously trained 2D CNN; Based on the type of the data and the required precision and the determination power for different axes, different neural networks may be used, however a single neural network trained with different part types is enough to determine the types of the sections. Using a neural network trained by two different types of 2D matrices, a type representing multi linear (partially or completely) sections (namely type “01”) and another representing circular ones (namely type “00”), the framework determines the type of each section as depicted in the last row of Fig. 13.

### Case 2

**Figure 14:**
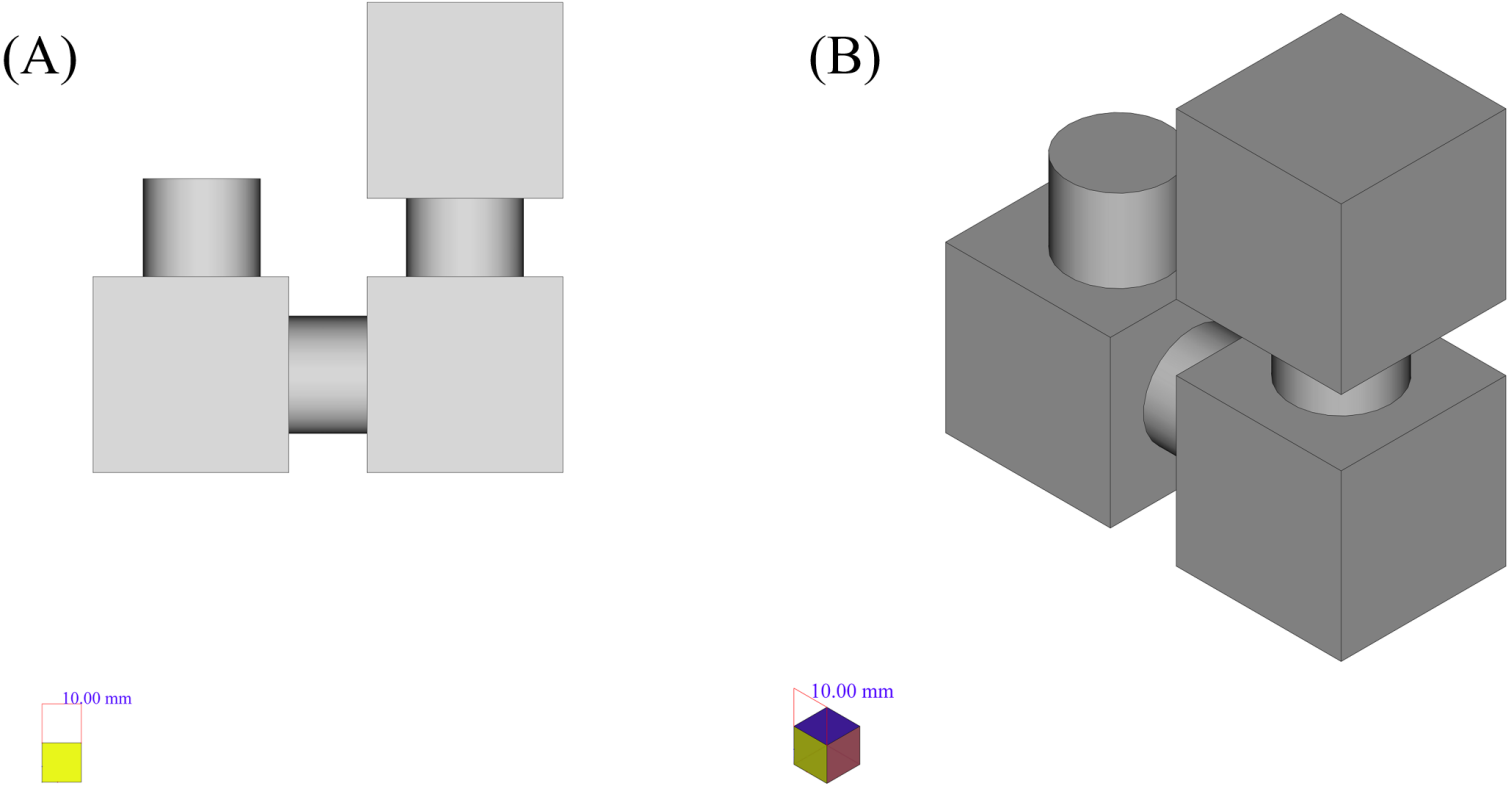
The initial shape of the example. (A) Side view. (B) Isometric view.

Another example that shows the applicability of the framework based on convex decomposition proposed in Appendix 1 is depicted in Fig. 14. This shape consists of 3 boxes and 3 cylinders connected to them. The reasons for choosing the shape in Fig. 14 are:

• It is a combination of many components and there are many cases in the real world where combinations of cylinders or cones and boxes, create the main outline of the shape.
• It can depict the power of the framework being within the limitations of the framework. In general terms, shapes which become problematic using the convex decomposition proposed in Appendix 1 include:

– Shapes which include sphere or torus or a part of them. The problem here happens due to the tessellated representation of these shapes consists of large number of facets and as the analysis in the convex decomposition algorithm is based on facets, this multitude of facets, facing many directions can cause unwanted cuts.
– Shapes consisting of the intersection of two (or more) shapes with curved cross sections. Here The problem happens due to the inseparability of these intersections using facets.

**Figure 15:**
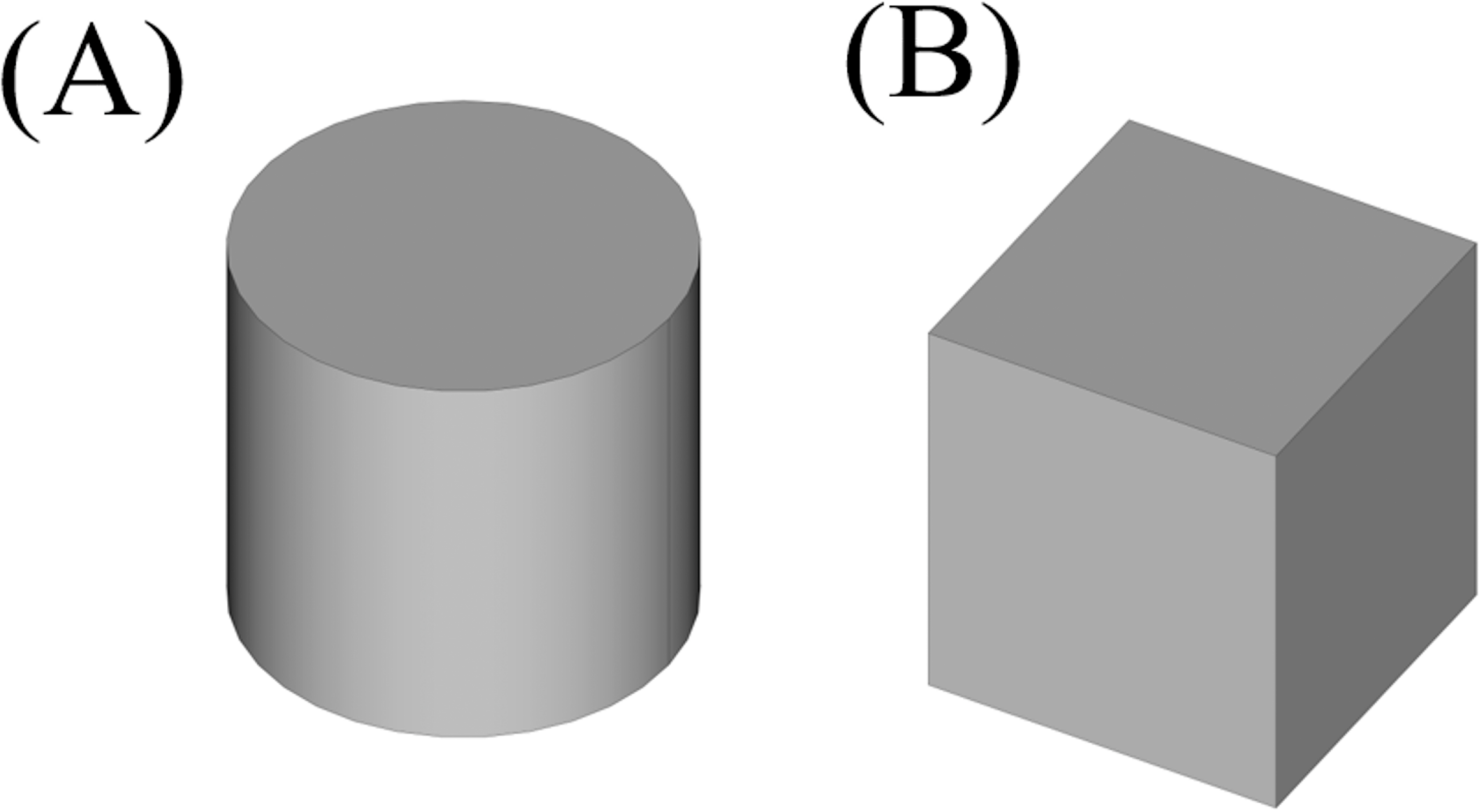
The resulted convex shapes from the convex decomposition of the initial shape. (A) Cylinder which connects the cubes (B) Cube.

The first step is to decompose this shape into its convex components. To do this the shape is tessellated to small planar triangles covering the shape. The resulted triangles would be accounted as one with each other with respect to their coplanarity, and the resulted planes would be used to decompose the shape into its convex parts respectively. Applying this method, the model decomposed the shape in Fig. 14 into separate shapes. As resulting shapes are similar, only two types of the resulted components are depicted in Fig. 15.

**Figure 16:**
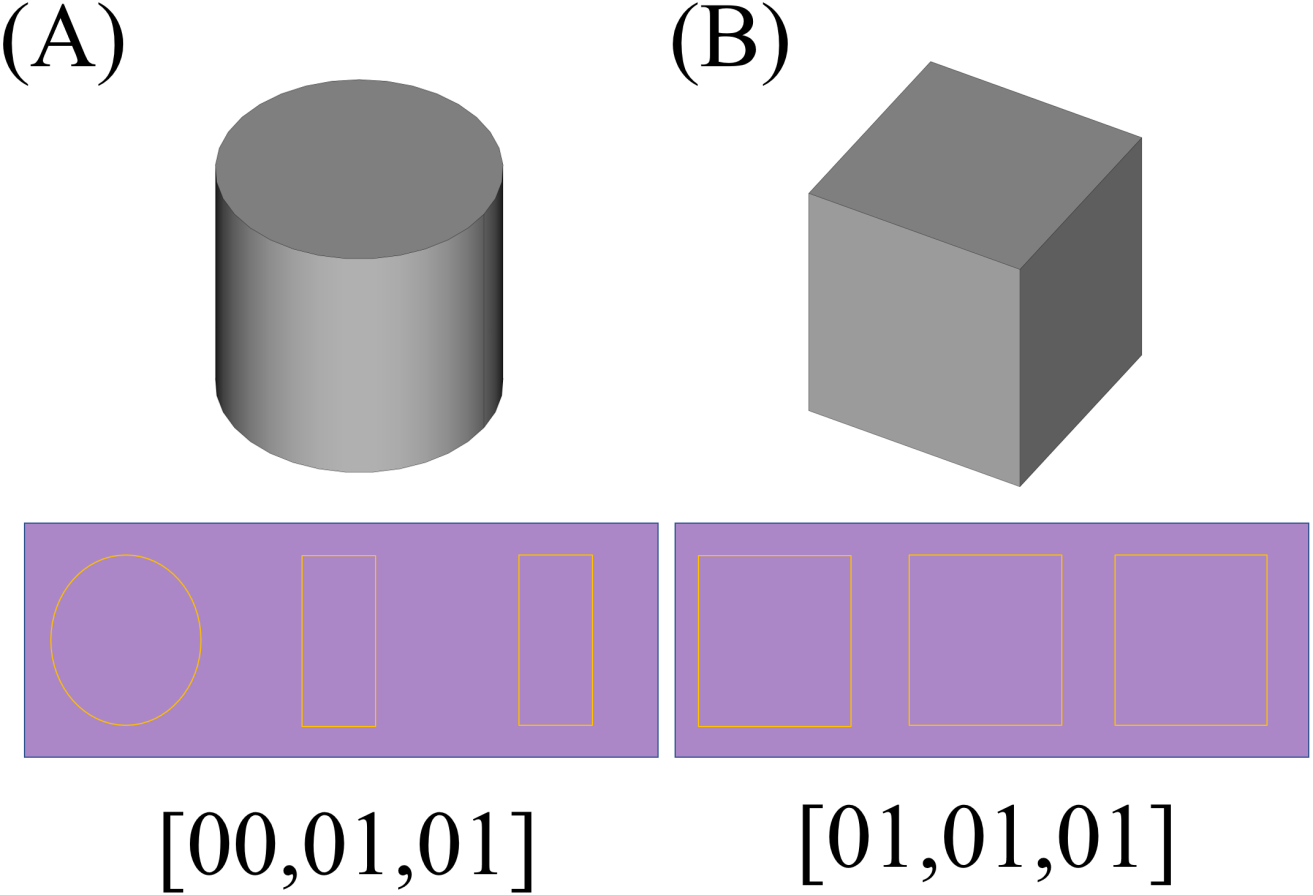
2D sections of each of the convex parts of the original shape. (A) 2D sections and the results of the recognition phase for each of the cylindrical components. (B) Results for each of the cubic components.

The resulted 2D sections for each shape is depicted underneath it in the Fig. 16 (like the previous example, the resulted matrices are depicted as 2D shapes in which the yellow pixels represent value 1 and other pixels represent value 0). By applying the recognition CNN applied in the previous case, the results would be as depicted in the last row of Fig. 16.

As mentioned before, the two fold framework proposed by this paper can be a preliminary step in micro and macro process planning of additive manufacturing products. The results of the categorizations, can be considered as features used by different viewpoints of process planning without need to define them explicitly; in fact, categories considered in the examples were intended to be simple cases, for the illustrative purpose of these examples. 2D CNN’s are one of the most powerful tools in the categorization of images (Tong, 2018) and by enriching their training data and some changes in their architecture, they are capable of categorizing a vast variety of images.

## Conclusion and Discussion

The cases analyzed in section “Case examples” are examples showing the power of the proposed framework in different regards including:

• Considering the input structure: the framework can perform the analysis using simple design information. In fact, the information provided in a 2D image of a symmetrical shape, taken from a viewpoint that shows the changes in the behavior of the outer edges of the shape is enough.
• Considering the decomposition phase: the decomposition is done, just using data provided in the shape itself and without need for additional training data. The geometric positions of the points on the shape is enough to decompose the shape.
• Considering the recognition phase: the recognition or categorization is accomplished using 2D CNN trained by data which represent simple attributes. The overall structure of the shape is represented by a combination of these simple attributes. This makes the preparation of the training data easier while representing a method to interpret more complex attributes as composition of simpler ones.
• Considering the application phase: each of the recognized curved or linear components can be manufactured applying special parameter level considerations based on their type (Zhao & Guo, 2020). From a higher level manufacturing technique viewpoint, some methods have better outputs facing curved surfaces (Zhang et al., 2020) and in a cloud platform where many techniques are available, such a framework can help to decide upon the technique autonomously.

In general terms, as it has been shown in section “Case examples” a divide and conquer viewpoint in the interpretation of complex shapes, can provide us with a powerful tool to interpret a vast variety of shapes. Regarding the recognition phase, although the power of convolutional neural networks is dominant, the simple categorization of the sections as to be completely circular versus partially or completely multi linear, is concerned and the outputs were in accordance with the expectations.

The framework proposed in this paper, creates a basement for an autonomous intelligent additive manufacturing system which can get a design in STL (or any other 3D design) file format, and interpret it as a union of some simpler shapes, regarding the desired geometrical attributes of which, the decision upon the production process can be made. In fact, the resulted categories would provide the system with information about the geometrical structure of the product that is of much concern in the process planning for the product.

One potential approach to expand this work is based on the learning capability: The recognition algorithm of this structure can be applied by using only two initial categories and some examples for each category; by the passage of time the data categorized by the neural network can be labeled based on the accuracy of categorization. The inaccurately categorized data can be braked into some groups by a clustering based method and when the data volume in each of the groups raises to a lower bound, these group of data be added to the training data of the neural network as a new category. Applying these steps, the proposed framework can learn new categories during its application.

**Figure 17:**
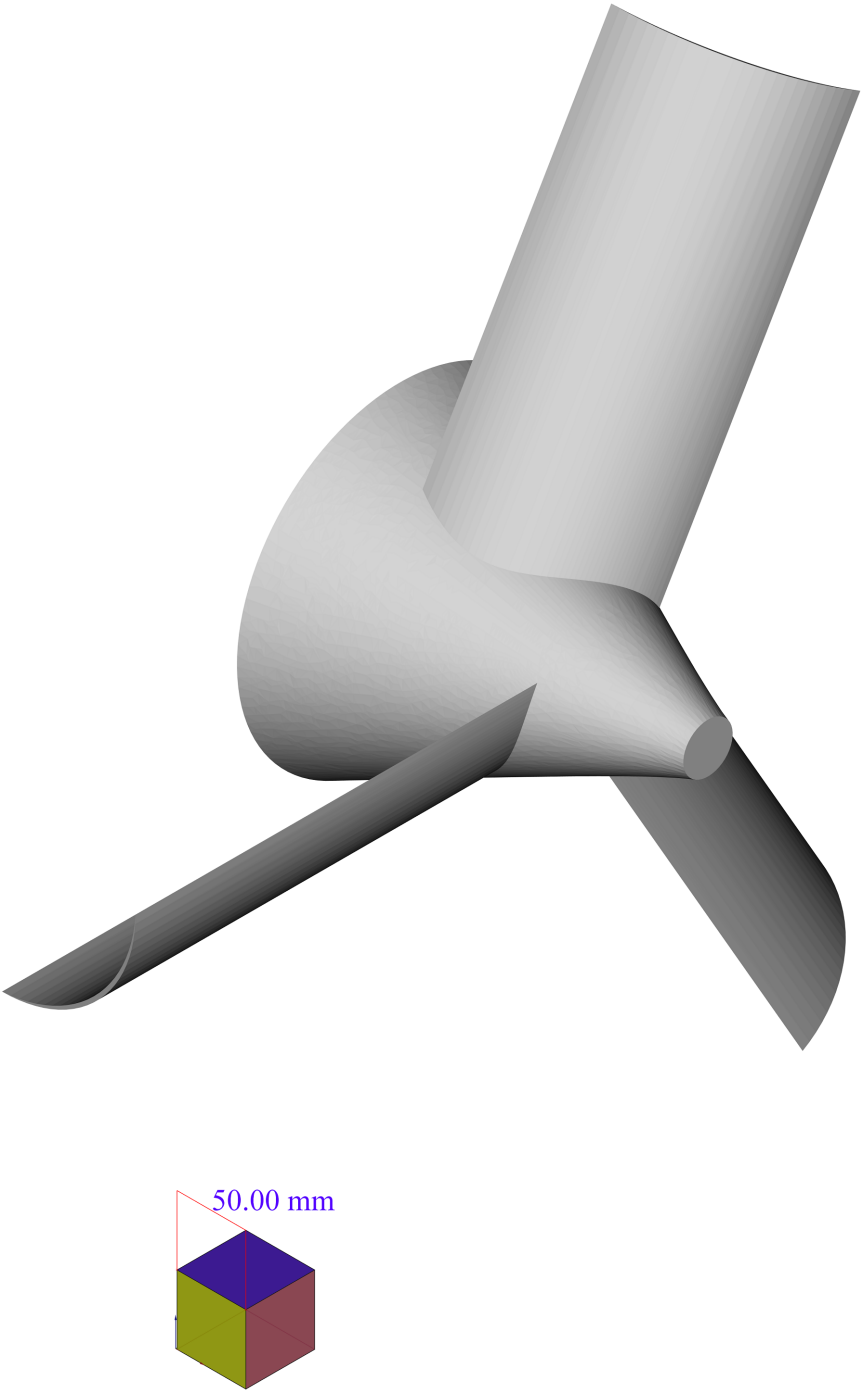
A simple propeller.

**Figure 18:**
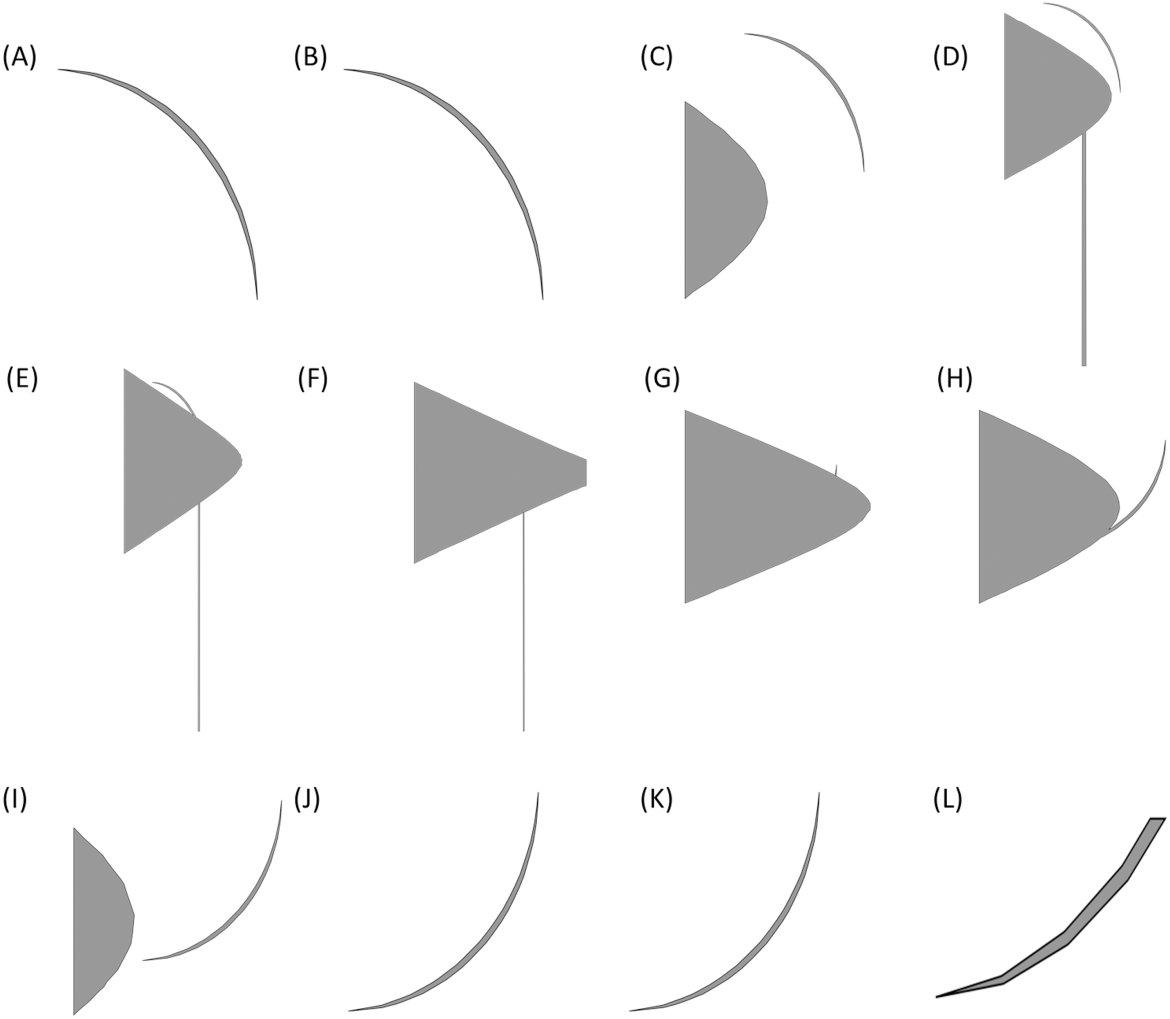
2D sections of the propeller. Pictures (A) to (L) show sections created by planes cutting the propeller in different heights.

To expand the shape decomposition part, it should be reminded that the proposed algorithm deals with simple shapes which meet the explained conditions. To improve the algorithm to be applicable to all types of shapes, two extensions are needed to be made:

• Improve the algorithm to be more sensitive to the changes in 2D sections images. The algorithm in the proposed form in Section 3, considers the changes to be only in the direction *z* of the Cartesian coordinate system (or in better words, by an accurate definition of the coordinate system, they would be so). However, in cases such as the propeller depicted in Fig. 17 there is no such a definition of the coordinate system that meets this condition.
• Apply the algorithm for the change detection not only to a single 2D section of the shape. Obviously, to deal with shapes with changes in different directions, most of the times, one 2D section is not enough. As an example consider the propeller of Fig. 17. Some of sections in this shape, which have been created by planes perpendicular to the *z* axis and parallel with *x* − *y* plane are as depicted in Fig. 18. These pictures show that these simple cuttings would not be enough to decompose the shape and detected changes in each section should be connected in a special manner to create accurate cutting planes to simplify the shape.

**Figure 19:**
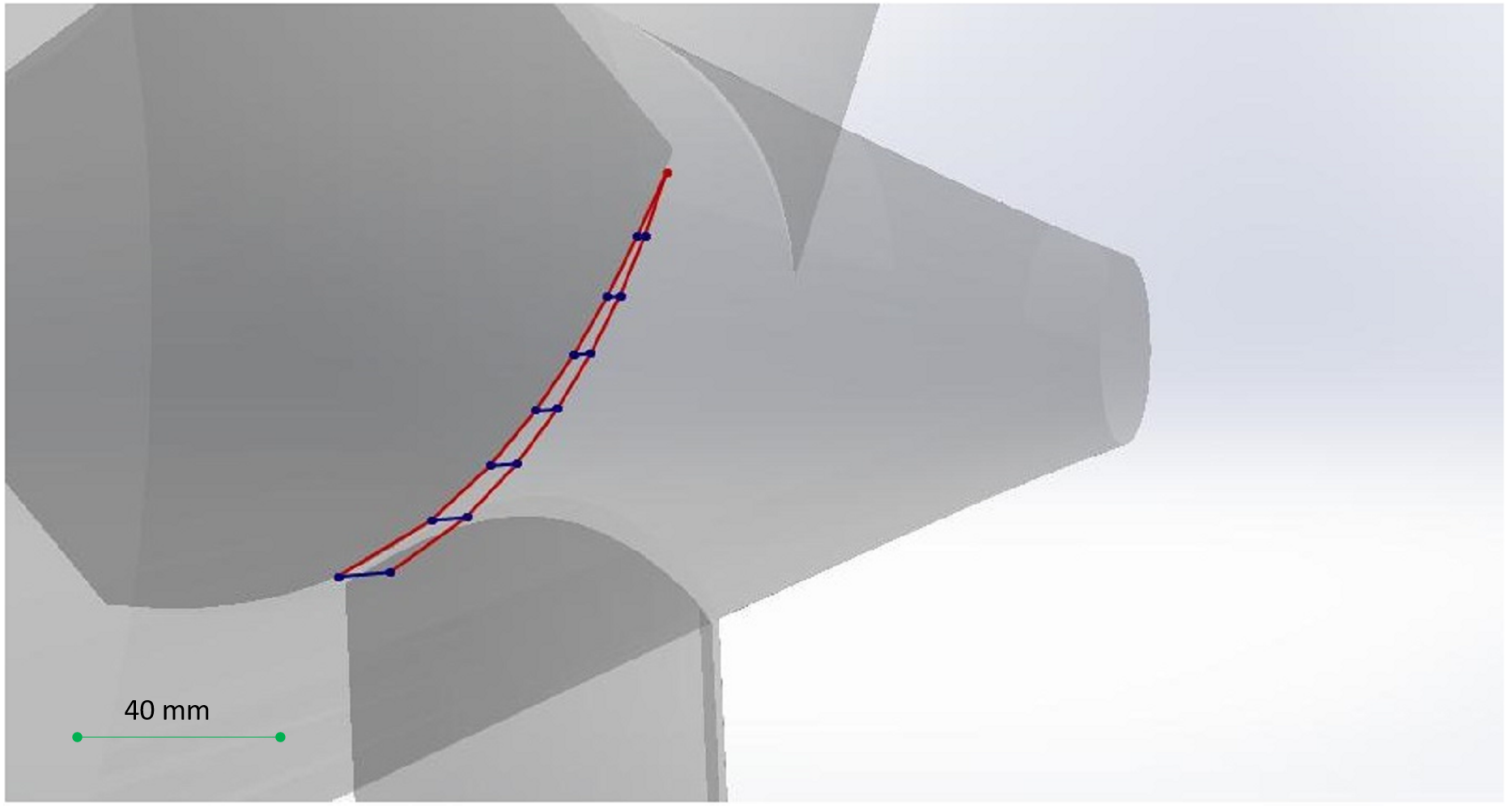
The semi-curved shape of the desired cut.

As an example of the desired output of the algorithm, suppose that we want to cut off one of the blades of the propeller. To illustrate the desired cut, consider Fig. 19. In Fig. 19 analysis in each of the perpendicular sections would result in the blue points (blue line segments show a section of the perpendicular section plane) and the red lines create the desired cut in 3D space (obviously with an error corresponding to the space between the lines as the behavior between two planes considered to be linear).

The expansion of the decomposition algorithm to solve such cases is the other related research potential. As the last proposed related research potential, it is recommended that the categorization outputs become inputs to a model, algorithm or another intelligent structure, to determine the production method based on the attributes of each category.

## Appendix A1. Convex decomposition as a simpler method for decomposition

Regarding the bounded randomness of the behavior of the neural networks (or other change detection methods), and considering the primitive based structure of the most products, in this appendix the convex parts of the shape are considered as the building blocks to be recognized. This point of view, creates a good intermediary level between fixed, predetermined basic features and the consideration of the shape as a whole because:

• The convex parts of the shape can be recognized using the information provided by STL (or other B-Rep) representations of the shape, using simple and general rules and assessments and there are many exact and heuristic methods to decompose the shape into its convex parts.
• Many convex parts in the real world are somehow modified versions of the basic CSG convex features. In fact, in many cases, the shape consists of some convex simple shapes that are similar to CSG convex features to a degree; but this similarity does not mean being limited to these features. The known building blocks in the proposed system can be expanded automatically by adding them to the dataset of the framework.
• From production point of view, the most of convex parts can be created by additive manufacturing using an accurate rotation without need for external base creation (complete sphere is an important exception).

As a result, at the first step, the shape is decomposed into its convex parts using the following algorithms. The “CDA” algorithm can substitute the data preparation and decomposition sections in Figs. 1 and 2:

Algorithm 4 Slicer Algorithm (namely ”SA”)

  1:  Get as an input, the shape of interest and name it S. 
  2:  Tessellate the faces of the input shape (reduce the faces into flat triangular facets of a pre- 
     determined maximum edge length).  Name the resulted shape TS and the set of the resulted 
     triangles STS. 
  3:  Find the set of maximal subsets of STS considering the coplanarity of the triangular facets. 
     Name the resulted set CSTS. 
  4:  For each element of the CSTS find the covering super plane using the normal vector and one 
     point of the plane (which are the normal and one point of one of its members respectively). 
     Name the set of resulting planes SP. 
  5:  for  each plane pl ∈ SP : do 
 6:       Create set SLP = {}. 
  7:       (Optional) If the plane pl slices S into at least two distinct shapes and doesn’t cut through 
     any edges of TS except in the vertices of the TS, add the resulted shapes into SLP; give SLP 
    as the output and finish the algorithm. 
  8:       If the plane pl slices S into at least two distinct shapes, add the resulted shapes into SLP; 
     give SLP as the result and finish the algorithm.

Algorithm 5 Convex Decomposition Algorithm (namely ”CDA”)

  1:  Get  the  input  shape  and  name  it  O.     Create  ordered  sets  Convexshapes  =   {}and 
     Concaveshapes = {O}. 
  2:  while the cardinality of the Concaveshapes set is not zero:  do 
 3:       Apply the SA algorithm on the first member of the Concaveshapes, which we name CV
    and name the result set RS. 
  4:       if  RS is not empty then 
 5:            Append the members of RS to the end of the Concaveshapes set. 
  6:       else 
 7:            Move CV from Concaveshapes set to Convexshapes set. 
  8:  Give Convexshapes set as the output.

## Additional Information and Declarations

### Data Availability

The following information was supplied regarding data availability: The developed software package is available at figshare: Fatahi Valilai, Omid (2021): 3D geometry intrepration. figshare. Software. https://doi.org/10.6084/m9.figshare.14016038.v1. The case studies are available at figshare: Fatahi Valilai, Omid (2021): 3D object samples. figshare. Figure. https://doi.org/10.6084/m9.figshare.14016122.v1.
